# Ultrasound-Guided Exercise Therapy Targeting the Supinator and Its Radial Nerve Branch for Refractory Lateral Elbow Pain: A Case Series

**DOI:** 10.7759/cureus.92970

**Published:** 2025-09-22

**Authors:** Shunta Shimizu, Masashi Kawabata, Masayoshi Saito, Toru Omodani

**Affiliations:** 1 Department of Rehabilitation, Tokyo Advanced Orthopaedics, Tokyo, JPN; 2 Department of Rehabilitation, Kitasato University School of Allied Health Sciences, Sagamihara, JPN; 3 Department of Orthopaedics, Tokyo Advanced Orthopaedics, Tokyo, JPN

**Keywords:** case report, lateral epicondylitis, radial nerve branch, refractory elbow pain, reverse thomsen test, supinator muscle, ultrasound-guided therapy

## Abstract

Lateral epicondylitis is commonly attributed to tendinopathy of the extensor carpi radialis brevis (ECRB). Although eccentric exercises, prolotherapy, and percutaneous ultrasonic tenotomy (PUT) are frequently used, a subset of patients experience persistent pain. The potential contributions of the supinator muscle and its radial nerve branch to rehabilitation have not been adequately explored. We report three patients (two females and one male; aged 20-59 years) with refractory lateral epicondylitis and residual pain after injection or PUT. All the patients exhibited severe pain during the modified Thomsen test in supination (“reverse Thomsen test”). Ultrasonography revealed impaired supinator activation with deep tenderness. Each patient underwent ultrasound-guided visual feedback training to selectively activate the supinator muscle, combined with a structured home exercise program. After 12-18 weeks of therapy (three to six sessions), pain resolved, tenderness subsided, and Disabilities of the Arm, Shoulder and Hand (DASH) scores improved significantly (Case 1: 25→9; Case 2: 48→4; Case 3: 15→4). These cases suggest that supinator dysfunction and involvement of the radial nerve branch may contribute to persistent lateral elbow pain. The reverse Thomsen test reliably reproduced the symptoms consistent with nerve stretching at the supinator level. Ultrasound-guided exercises facilitated selective supinator recruitment, reduced compensatory extensor overactivity, and improved functional outcomes. These findings indicate that supinator-focused rehabilitation may be a valuable therapeutic option in refractory cases. Ultrasound-guided exercise therapy targeting the supinator and its radial nerve branches provided substantial clinical benefits to patients with refractory lateral epicondylitis. A positive reverse Thomsen test result may serve as a clinical indicator of supinator involvement and help guide treatment selection.

## Introduction

Lateral epicondylitis, commonly referred to as “tennis elbow,” is a prevalent musculoskeletal disorder characterized by pain at the lateral epicondyle [1,2]. It is primarily associated with tendinopathy of the extensor carpi radialis brevis (ECRB), which is triggered by repetitive wrist extension and forearm supination [1]. Pain is typically reproduced with extensions of the resisted wrist or middle finger [2]. Its prevalence is estimated to be 1-3% in the general population and is particularly high among individuals engaged in heavy manual labor [2].

Conservative treatments, including rest, activity modification, therapeutic exercise, manual therapy, bracing, and pharmacological interventions, are generally recommended [1,3]. When symptoms persist, corticosteroid injections or percutaneous ultrasonic tenotomies (PUT) are often considered [4,5]. Exercise therapy with eccentric contraction has shown superior short-term benefits compared with concentric or isometric exercises [6]. However, patients complicated by neuropathic involvement or chronicity often respond poorly to conventional rehabilitation [7,8].

Although injections and surgical interventions may relieve the symptoms in some patients, others experience persistent pain, particularly during wrist extension and supination. Simon et al. have reported that radial tunnel syndrome and lateral epicondylitis may be difficult to differentiate, and that when these two conditions coexist, both should be managed concurrently [9]. Notably, few reports have addressed rehabilitation approaches focusing on the supinator muscle and its radial nerve branch, despite their anatomical relationship with pain provocation in lateral epicondylitis.

Herein, we describe the cases of three patients with refractory lateral elbow pain who experienced persistent pain after injection or PUT. Ultrasonographic evaluation revealed impaired contraction of the supinator muscle with compensatory overactivation of the ECRB and extensor digitorum. We applied ultrasound-guided exercise therapy specifically targeting the supinator and its radial nerve branch, which resulted in significant symptom relief.

## Case presentation

Patients

**Figure 1:**
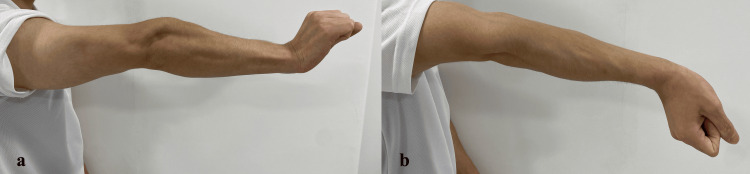
Patient positioning for the reverse Thomsen test (a) Thomsen test (b) reverse Thomsen test

**Figure 2:**
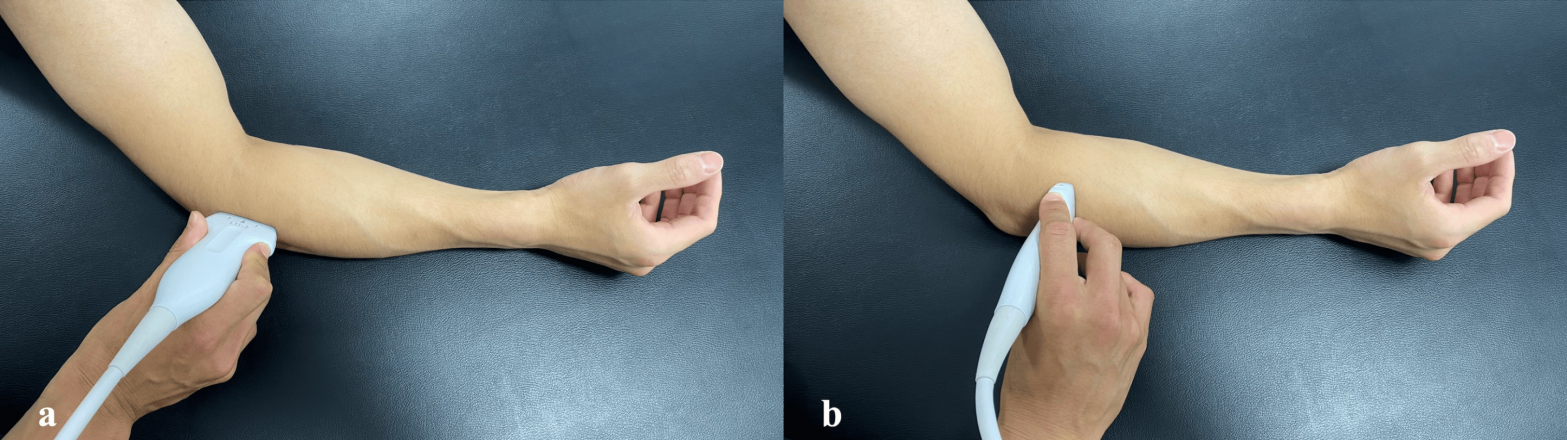
Probe position (a) Long-axis placement for evaluating the common extensor tendon attachment. (b) Short axis placement to assess dynamic function of the supinator muscle.

**Figure 3:**
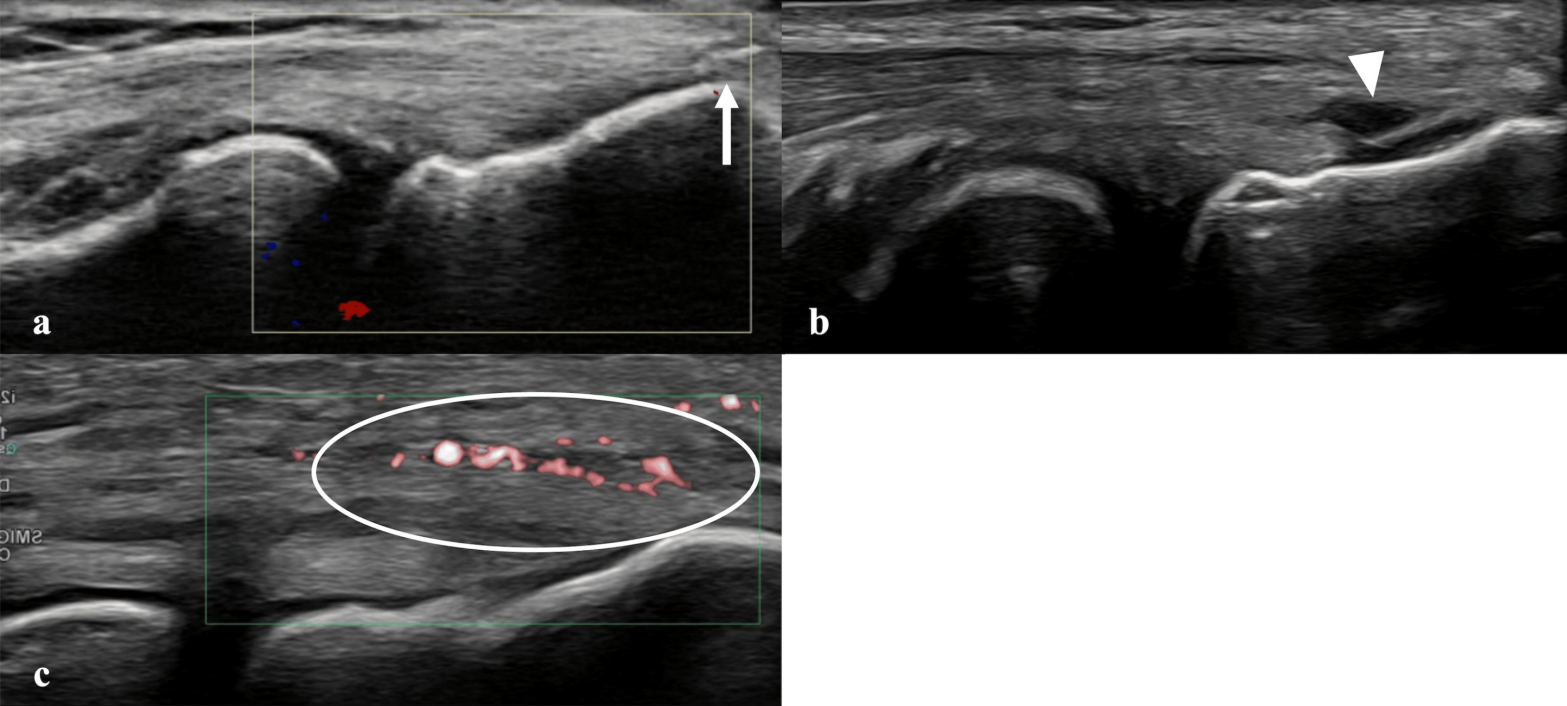
Ultrasonographic findings at the tendon insertion (a) Hyperechoic lesion at the lateral epicondyle suggestive of calcification (white arrow). (b) Hypoechoic lesion within the tendon suggestive of a partial tear (white arrowhead). (c) Tendon swelling and increased vascularity demonstrated by enhanced Doppler signal (white circle).

Three patients (two females, one male; aged 20-59 years) with chronic lateral epicondylitis were included. All patients exhibited positive findings on a modified Thomsen test performed in forearm supination (“reverse Thomsen test”) (Figure 1). Detailed characteristics are summarized in Table 1. At baseline, the Disabilities of the Arm, Shoulder, and Hand (DASH) scores were 25 (Case 1), 48 (Case 2), and 15 (Case 3). Upper extremity function was evaluated using the DASH questionnaire, including the optional sports module when applicable. The DASH was originally developed by Hudak et al. [10], and the Japanese version was validated by Imaeda et al. [11]. Permission for its use in this non-commercial academic study was obtained in compliance with the copyright policy of the Institute for Work & Health (Toronto, Canada), the copyright holder. As noted on the institute’s website (https://dash.iwh.on.ca/), a formal license agreement was not required. Ultrasound findings demonstrated tendinopathic changes at the common extensor origin in all cases (Figures 2, 3). Case 2 had previously undergone prolotherapy, and Case 3 had undergone PUT.

**Table 1:** Case summary PT, physical therapy; PUT, percutaneous ultrasonic tenotomy

Case	Age/sex	Duration of disease	Course until the start of physical therapy	Treatment period / number of treatments
					#1	40s / Female	1 month	Physical therapy was initiated at the first visit without injection.	18 weeks / 6 PT sessions, 2 injections
										#2	50s / Female	2 months	At the first visit, an injection using hypertonic glucose solution into the extensor carpi radialis brevis tendon (prolotherapy) was administered, and physical therapy was started on the same day.	12 weeks / 3 PT sessions, 1 injection
#3	40s / Male	1 year	At another hospital, prolotherapy and steroid injections were performed, but no clear improvement was observed. The patient visited our hospital and underwent PUT one week after the initial consultation. Physical therapy was started the following week.	13 weeks / 6 PT sessions, 1 PUT										

Clinical findings

The pain persisted even after medical intervention. All the patients experienced marked pain during the reverse Thomsen test (forearm supination). Ultrasonography revealed insufficient activation of the supinator during forearm supination compared with the contralateral side (Video 1). Localized tenderness was observed within the supinator region, particularly in the deep portion adjacent to the radial neck.

**Video 1:** Ultrasonographic evaluation and muscle contraction dynamics during forearm supination before therapy (short-axis view) (a) The forearm is supinated from the neutral position through assisted active movement with the elbow in extension. The wrist is maintained in slight passive extension. (b) Insufficient supinator contraction was qualitatively assessed and indicated by the magnitude of muscle thickness change.

Intervention

Ultrasound-guided visualization of both the short- and long-axis images of the supinator was performed. To facilitate deep contraction of the supinator, manual manipulation targeting the supinator branch was applied to the deep portion of the supinator, combined with repeated supination movements. Contraction was confirmed by straightening the muscle fibers. Real-time visual feedback was provided to reinforce selective activation of the supinator while minimizing compensatory activity of the wrist extensors (Video 2).

**Video 2:** Manual manipulation and exercise therapy targeting the supinator and its radial nerve branch (a) Position for manual manipulation and exercise: With the elbow slightly flexed, the supinator was pressed toward the radius using the examiner’s fingers, followed by repeated voluntary contractions of the supinator. (b) Ultrasonographic recording. Manual manipulation was performed to mobilize the radial nerve branch coursing through the deep portion of the supinator (white arrow). Repeated contractions confirmed recruitment, extending to the deep fibers of the supinator.

**Figure 4:**
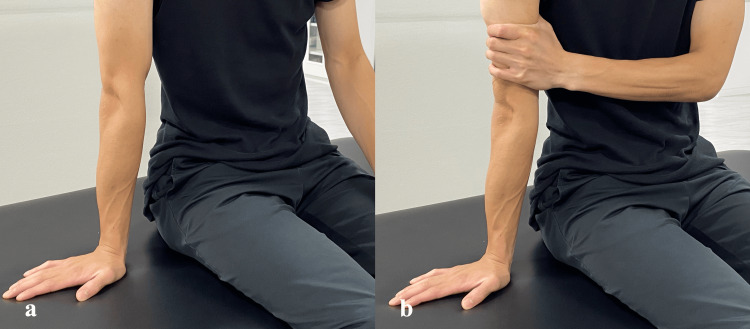
Positioning for the home exercise program (a) Starting position. (b) Isometric forearm supination performed with the shoulder internally rotated and the wrist maintained in passive extension.

In addition, patients were instructed to perform a structured home exercise program, with the shoulder internally rotated and the wrist maintained in passive extension; the forearm was isometrically supinated to place the wrist extensors in a shortened position and preferentially recruit the supinator (Figure 4). Patients were instructed to perform this program once daily, consisting of three sets of 10-15 repetitions, accompanied by visual feedback to ensure selective supinator contraction. Ultrasonography was employed by the physiotherapist during the supervised sessions to provide visual feedback to the patients. The patients did not operate the ultrasound device at home; rather, the home exercise program was carried out based on the verbal instructions and visual feedback they had received during the clinical sessions.

Outcomes

**Figure 5:**
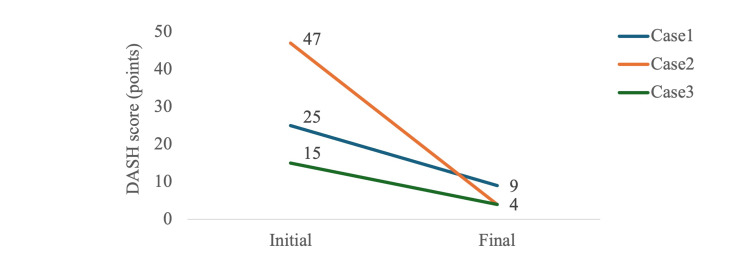
Changes in DASH scores at baseline and post-treatment DASH: Disabilities of the Arm, Shoulder and Hand

After 12-18 weeks of therapy (three to six supervised sessions), all the patients exhibited substantial clinical improvement. Ultrasound evaluation confirmed diminished compensatory activation of the wrist extensors and restoration of selective supinator contractions (Video 3). Pain elicited by the reverse Thomsen test decreased immediately after the initiation of therapy and was completely resolved by the end of treatment (Table 2). This table summarizes the findings obtained at the final treatment session. Functional outcomes also improved markedly, with DASH scores decreasing from 25 to 9 in Case 1, 48 to 4 in Case 2, and 15 to 4 in Case 3 (Figure 5). No adverse events were observed during the study period.

**Video 3:** Changes in muscle contraction dynamics during forearm supination before and after therapy (a) Prior to therapy, supinator contraction was insufficient with compensatory activation of the extensor carpi radialis brevis (ECRB) and extensor digitorum. (b) After therapy, compensatory contraction of the ECRB and extensor digitorum is reduced, with selective improvement in supinator contraction.

**Table 2:** Clinical findings and outcomes in each case Forearm supination and wrist extension strengths were assessed using the Manual Muscle Testing (MMT) scale (range: 0–5). Symbols: (+) mild; (++) moderate; (±) slight; (−) none. *: Pain-associated muscle weakness (assessed with the elbow in extension).

Case / clinical findings	Thomsen test		Reverse Thomsen test		Forearm supination strength		Wrist extension strength		Sensory disturbance	
	Pre	Post	Pre	Post	Pre	Post	Pre	Post	Pre	Post
Case 1	+	−	++	−	4	5	4*	5	−	−
Case 2	+	−	++	±	4	5	4*	5	−	−
Case 3	+	−	++	−	4	5	4*	5	−	−

## Discussion

This case series highlights the potential role of the supinator and supinator branch of the radial nerve in refractory lateral epicondylitis. Despite conventional medical interventions (prolotherapy or PUT), all the patients experienced persistent pain that was reproduced by the reverse Thomsen test and localized to the supinator using ultrasound guidance.

In a double-blind randomized controlled trial, Scarpone et al. reported that prolotherapy for refractory lateral epicondylitis resulted in significant pain reduction at 16 weeks compared with saline injection [12]. Similarly, in a randomized trial of chronic lateral epicondylitis, Peterson et al. demonstrated that eccentric exercise achieved greater pain reduction than concentric exercise at eight weeks [13]. However, these benefits were not universal; approximately 40% of patients in the prolotherapy group still had symptoms at 52 weeks, and approximately 20% of those in the eccentric exercise group failed to achieve sufficient improvement [12,13]. Furthermore, although nearly 80% of the patients with lateral epicondylitis recover spontaneously within one year of diagnosis, those with comorbidities, particularly radial tunnel syndrome, often respond poorly to conservative treatment and experience more persistent symptoms [8,14]. These findings underscore the complexity of pain mechanisms in lateral epicondylitis and the need for alternative treatment strategies.

Arrigoni et al. reported that repetitive varus and pronation loading of the elbow may lead to elongation of the lateral collateral ligament, resulting in abnormal radial head translation [15]. Furthermore, Nimura et al. demonstrated that the lateral collateral ligament can be interpreted as a structure integrated with the joint capsule and the supinator aponeurosis, which collectively contribute to the lateral stability of the elbow [16]. Accordingly, the supinator can be considered a key muscle for maintaining lateral stability. On the other hand, repetitive varus and pronation loading may also occur during daily activities, such as pouring a drink or performing office work [15]. This repetitive stress may force the supinator to undergo repeated eccentric contractions, predisposing it to hypertonicity.

Anatomically, the supinator comprises superficial and deep heads that encircle the proximal radius [17]. Biomechanical studies have shown that when the wrist is placed from a neutral position into flexion-pronation, the radial tunnel pressure increases. This elevated pressure can be reduced by 77% following tenotomy of the supinator superficial head, indicating that the superficial head plays a major role in pressure elevation [18].

These findings suggest that supinator hypertonicity may also contribute to radial nerve entrapment and that its reduction is an important therapeutic target in physical therapy. The posterior interosseous nerve passes beneath the superficial head, and the supinator branch of the radial nerve extends posteriorly around the radial neck, supplying the posterior elbow capsule and proximal radioulnar joint [19]. This branch corresponds to the region where tenderness was observed on ultrasonography. Supination stretches the nerve at this level, which explains the pain elicited during the reverse Thomsen test. Furthermore, pain provocation with resisted supination during elbow extension is a recognized feature of radial tunnel syndrome, supporting the involvement of neural structures [20].

Our findings suggest that supinator dysfunction, characterized by reduced recruitment and tenderness at the deep portion, may lead to a compensatory overload of the ECRB and extensor digitorum, perpetuating tendon pain at the lateral epicondyle. Ultrasound-guided selective activation of the supinator enables neuromuscular re-education, reduces compensatory patterns, and alleviates pain. These results support the clinical utility of targeting the supinator and its radial nerve branch in the rehabilitation of refractory lateral epicondylitis, particularly in patients with a positive reverse Thomsen test.

This study had some limitations. First, the evaluation of supinator contraction was qualitative; quantitative electromyography or strength measurements were not performed. Second, the relationship between the improved supinator function and reduced mechanical stress at a common extensor origin remains unclear. Third, owing to the small size of this case series, the obtained results require confirmation via larger controlled studies. In addition, the absence of a control group means that potential placebo effects and the natural course of the condition cannot be excluded.

## Conclusions

In summary, ultrasound-guided exercise therapy targeting the supinator and its radial nerve branch appeared to reduce pain and improve function in patients with refractory lateral epicondylitis. The reverse Thomsen test may help identify supinator involvement and guide rehabilitation strategies. Although this study is limited by its small sample size and the qualitative nature of muscle activity assessment, the findings suggest potential clinical utility and warrant further investigation in future studies.
